# Implicit Measures Help Demonstrate the Value of Conservation Education in the Democratic Republic of the Congo

**DOI:** 10.3389/fpsyg.2020.00386

**Published:** 2020-03-13

**Authors:** Aleah Bowie, Christopher Krupenye, Pierrot Mbonzo, Fanny Minesi, Brian Hare

**Affiliations:** ^1^Department of Evolutionary Anthropology, Duke University, Durham, NC, United States; ^2^School of Psychology and Neuroscience, University of St Andrews, St Andrews, United Kingdom; ^3^Lola ya Bonobo, Les Amis des Bonobo, Kinshasa, Democratic Republic of the Congo; ^4^Center for Cognitive Neuroscience, Duke University, Durham, NC, United States

**Keywords:** conservation, education, bonobo, Congo, great apes, Central Africa

## Abstract

Biodiversity is being lost at unprecedented rates. Limited conservation resources must be prioritized strategically to maximize impact. Here we introduce novel methods to assess a small-scale conservation education program in the Democratic Republic of Congo. Lola ya Bonobo is the world’s only sanctuary for one of humans’ two closest living relatives, bonobos, orphaned by the illegal trade in bushmeat and exotic pets. The sanctuary is situated on the edge of the country’s capital, Kinshasa, its most densely populated region and a hub for the illegal wildlife trade that is imperiling bonobos and other endangered species. Lola ya Bonobo implements an education program specifically designed to combat this trade. Previous evaluation demonstrated the program’s efficacy in transmitting conservation knowledge to children. In Study 1, we use novel implicit tests to measure conservation *attitudes* before and after an educational visit and document a significant increase in children’s pro-conservation attitudes following direct exposure to bonobos and the education program. In Study 2, we show that adults exhibit high levels of conservation knowledge even before visiting the sanctuary, likely due to the sanctuary’s longstanding education efforts in Kinshasa. In Study 3, we explored adults’ empathetic attitudes toward bonobos before and after the sanctuary tour. Our results support the conservation education hypothesis that conservation education has improved relevant knowledge and attitudes in Kinshasa. Crucially, the present study validates new methods for implicitly assessing attitudes about environmental and social issues. These methods overcome typical biases in survey sampling and can be employed in diverse populations, including those with low literacy rates.

## Introduction

Overwhelming scientific evidence points to severe threats against our planet’s ability to sustain high levels of biodiversity. Human population growth, climate change, industrialization and many other forces are all working in concert to drive an exponential increase in species extinction (Ceballos et al., 2017). One of the main tools utilized to combat extinction is environmental education (Bickford et al., 2012). Many international non-profit organizations have invested tremendous time and effort into providing educational resources to encourage conservation efforts among their target populations (Stern et al., 2014; Thomas et al., 2019). The *conservation education hypothesis* (CEH) suggests that people are more likely to defend conservation if they have been exposed to knowledge about endangered species and ecosystems (Bickford et al., 2012; Ardoin et al., 2018). This hypothesis would predict that a change in knowledge about a given species, or familiarity with facts about the ecology, biology, conservation threats, and conservation status of a given species or its ecosystem, also leads to positive changes in attitudes toward them. Pro-conservation attitudes are ways of thinking or feeling that support the welfare and survival of a given species or ecosystem. The null hypothesis in this case suggests that most environmental education programs do little to change attitudes at a scale that can have a significant impact (Struhsaker et al., 2005). In this case, priority investments should be made in policies and actions that directly protect habitat or threatened environments over education programs (Ferraro and Pattanayak, 2006). Testing the predictions of the CEH is increasingly important as communities, governments and non-profits try to determine how best to allocate finite resources.

A key test of the CEH involves evaluating existing education programs. Many conservation education programs survey individuals before and after their educational experience (Monroe et al., 2017). The prediction in these pre-experience/post-experience surveys is that the participants will show higher levels of knowledge and more positive attitudes toward conservation afterward. The advantage of this assessment approach is that it is easy to implement in a variety of settings and is relatively inexpensive. Survey evaluations have been able to identify programs that effectively communicate their message, optimize existing programs, and detect programs that are not effective (Kruse and Card, 2004; Cutter-Mackenzie and Smith, 2010). Various non-profit organizations are increasing their use of such survey assessments to demonstrate the impact of their education programs. However, even with more assessments, there is skepticism regarding the value of small-scale education programs – particularly those implemented across cultures (Carleton-Hug and Hug, 2010; Cutter-Mackenzie and Smith, 2010; Braun et al., 2018; Briggs et al., 2019). There is concern that effective education programs cannot realistically reach the increasing population sizes in areas surrounding vulnerable wildlife populations (Struhsaker et al., 2005). Reviews of these programs have suggested that given the costs of these conservation education programs, a net positive impact of conservation education may not exist at a more macro-level (Struhsaker et al., 2005; Ardoin et al., 2018).

The uncertainty of the impact of education programs underscores the importance of assessment and refinement. It also raises the question of which techniques are best for evaluating conservation education. Traditionally education program surveys *explicitly* ask questions about attitude toward conservation. However, decades of research on human cognition suggest that explicit questions of attitude are likely to be influenced by experimenter demand effects and answers may not be related to the actual internal preferences of the individual assessment-taker (Kintz et al., 1965; Cunningham et al., 2004; Nosek, 2005). If a tour is led by a conservationist at a conservation site, participants may be inclined to answer “yes” if asked “do you think more effort should be invested in this species’ conservation” even if it’s not how the participant truly feels. The results of explicit questions of attitude may therefore overestimate the pro-conservation attitudes of participants in conservation education programs.

Another methodological impediment for conventional pre–post experience evaluations is that they often rely on written surveys that can only be used with literate population. This precludes surveying large portions of the adult and child populations in many biodiversity hotspots around the world like Central Africa, Southeast Asia and the Amazon where childhood education is not universal (Jha and Bawa, 2006). It is with these populations, however, that NGOs are increasing their focus on sustainable development and conservation initiatives (Jha and Bawa, 2006; Bradshaw et al., 2015). Effective biodiversity conservation also often relies on changes in knowledge, attitudes and behaviors of multiple populations across several linguistic, ethnic and national lines (Briggs et al., 2019). Language translation often makes comparing the effectiveness of conservation programs across different cultures difficult (Evans et al., 2007; Briggs et al., 2019). Developing evaluation techniques that do not rely heavily on reading or writing will facilitate comparative evaluations since they can be implemented across cultures, therein helping organizations to develop programs that have the greatest impact on larger scales.

To test the CEH, we designed a pair of surveys for use at the Lola ya Bonobo sanctuary. Located in Kinshasa, a city of over 10-million citizens and the capital of the Democratic Republic of Congo, Lola ya Bonobo is the world’s only sanctuary for orphaned bonobos (*Pan paniscus*). Lola ya Bonobo functions as the only venue in the capital for adults, children, and governmental decision makers to observe and learn about great apes in person. This function is particularly vital because the Democratic Republic of Congo is home to the largest remaining populations of wild apes in Africa, including the world’s only bonobos as well as populations of chimpanzees and gorillas. Both bonobos and chimpanzees (*Pan troglodytes*) are humans’ closest living genetic relatives (Prüfer et al., 2012). The sanctuary provides high quality life-time care to wild-born bonobos that have been rescued from the illegal hunting and pet trades. The bonobos arrive in variable states, sometimes physically distressed—malnourished and riddled with parasites—as a result of the improper captive living conditions from which they have been rescued. They are also sometimes psychologically traumatized, having been separated from their mothers and their natural habitat (Wobber and Hare, 2011). Once they have arrived at the sanctuary, young infant orphans are provided with specialized care to help them overcome the acute trauma of their capture from the wild (Wobber and Hare, 2011). They are first cared for by substitute human mothers who help provide the first steps of rehabilitation. After 1–2 years with the substitute mothers, they are gradually integrated into peer groups where they enjoy rich social lives in large forested enclosures similar to what they would experience in the wild. Ultimately, the vast majority of bonobos experience a full recovery, living species-typical lives, exhibiting species-typical behavior and cognition (Wobber and Hare, 2011), and sometimes even being released back into the wild.

Six days a week, the sanctuary’s education team provides guided tours around the sanctuary for national and international visitors. Tens of thousands of children, adults and civil servants are exposed to the natural behavior of the highly charismatic bonobos while learning about their natural history and the threats to their survival in the wild. This includes information about the importance of the Congo Basin for the health and wellbeing of the people who live there as well (André et al., 2008).

**FIGURE 1:**
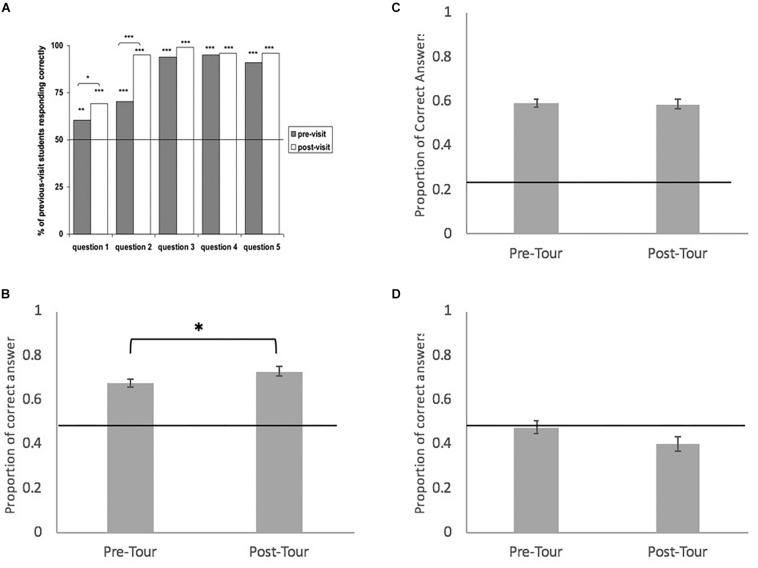
Results for conservation knowledge and attitude assessments among children and adults in the DRC. **(A)** (Reprinted with permission from Springer/Nature) results from André et al. (2008)’s knowledge assessment with percentage of correct responses to the following T/F questions: (1) Bonobos do not make good pets; (2) Bonobos are not an endangered species; (3) Hunting and snares are dangerous for bonobos; (4) Planting trees is something you can do to help bonobos; and (5) The bushmeat trade threatens bonobos with extinction. All participants were children visiting the sanctuary for the first time, and participants did significantly better on all five questions after the tour compared to before the tour. **(B)** Displays the mean proportion of correct answers in the pre-tour and post-tour Attitude Assessment. Overall, the mean proportion of correct answers were significantly higher in the post-tour condition than in the pre-tour condition (estimate = 0.27, *p* = 0.006). **(C)** Displays the proportion of correct answers for the pre-tour and post-tour Knowledge assessment. There was no difference between means on the two assessments, and the mean overall proportion of correct answers for both conditions were above chance. **(D)** Displays results for the Empathy assessment. There was no difference between overall scores and means for both assessments were below chance. **(B–D)** All display standard error.

In 2009, Lola ya Bonobo conducted a survey with 400 Congolese children to assess the education program’s success in transmitting conservation knowledge (André et al., 2008). All children took a knowledge assessment before and after participating in the education program. Half of the participants had never visited the sanctuary before, and half had done so 1 year earlier. In the pre-test, first-time visitors scored at or below chance on all questions whereas return children scored above chance on the majority of questions. In the post-test, children of both groups scored at ceiling on all questions (Figure 1A). This study shows that the sanctuary’s education program not only successfully teaches children key facts about conservation but also that the majority of what they learn is retained for at least a year.

This previous study also briefly assessed children’s explicit attitude toward bonobos. Participants were also asked if they found bonobos amusing, scary, dangerous, or beautiful. Less than 10% of participants described bonobos as amusing before their first tour whereas nearly 90% did so after observing bonobos at the sanctuary. While this explicit attitude assessment was limited to a single question it appears a similar pattern may also apply to the positive feeling children attribute to bonobos after their visit.

Building on this work, in the current study we test the CEH with two additional assessments of the education program at Lola ya Bonobo (Figure 1 and Table 1). In Study 1, we use novel implicit methods to measure changes in children’s conservation attitudes in response to the education program. These picture-based methods minimize experimenter-demand effects and do not require that participants can read or write. In Study 2, we investigate the education program’s ability to improve conservation knowledge of adult visitors, as adults are the primary decision-makers involved in conservation policy. In Study 3, we explore whether the education program has an effect on adult visitors’ empathy toward bonobos. For all of these studies, we predicted that in accordance with the CEH, participants in our experiments will show higher levels of knowledge and more positive attitudes toward bonobo conservation after participating in an educational visit to the sanctuary. Though the ultimate goal of conservation education is to encourage long-term behavior change that benefits the welfare of wildlife species, assessing the impact of programs on behavior change is notoriously difficult. Given the lack of studies of any type on conservation education in *in situ* programs in the Democratic Republic of the Congo, this study provides ample assessment of programs impact on knowledge and attitude changes in a population, which will help pave the way to study long-term behavior changes in the future.

**TABLE 1:** Rationale for the series of surveys in Studies 1-3 examining conservation attitudes and knowledge in children and adults.

	Child	Adult
Knowledge	André et al., 2008 Does the tour of the sanctuary increase children’s knowledge and understanding of bonobos and their conservation status?	*Study 2: Knowledge Assessment* Does the tour of the sanctuary increase adults’ knowledge and understanding of bonobos and their conservation status?
Attitude	*Study 1: Attitude Assessment* Does the tour of the sanctuary impact children’s attitudes toward bonobos and their conservation?	*Study 3: Empathy Assessment* Does the tour of the sanctuary affect adults’ empathic attitude toward bonobos?

**Previous research found that the education program at Lola Ya Bonobo had a significant impact on children’s knowledge relevant to bonobo conservation (André et al., 2008). The current research extends this work by assessing how the same program impacts children’s attitudes toward bonobos, as well as how the program impacts knowledge and attitudes of adults.**

## Study 1: Attitude Assessment

In this study we extend the André et al. (2008) assessment of Lola ya Bonobo’s conservation education program by again surveying children before and after they visit the sanctuary. However, to do so, we introduce novel implicit measures to assess participants’ conservation attitudes. The assessment was designed to appear to participants as if we were requesting their input for new designs for publicity for the sanctuary. Because participants are not made explicitly aware that they are being asked about their attitudes toward bonobos and their habitat, these measures are able to overcome experimenter demand effects and, therefore, should more honestly reflect participants’ conscious or unconscious beliefs about conservation issues. Consistent with the conservation education hypothesis, we predict that educational visits to the sanctuary will improve conservation attitudes.

### Attitude Assessment Methods

Participants were grade school students of Congolese origin attending one of four schools in Kinshasa (*N* = 203, mean age = 12.39, range = 7–19 years, M/F = 97/96). Two of the schools (Kimbala and Mamfufu) were in relatively rural regions outside the city and the other two schools (Nova Eligio and Ngolu) were in urban areas in the city center. None of the participants had previously visited the sanctuary. Experimental instructions were explained to the students by a familiar teacher.

The Attitude Assessment contained twelve questions that implicitly examined whether participants held pro-conservation or non-conservation attitudes (see Supplementary Material). Photos, instead of text, were used to control for literacy levels among participants. Each participant was given an assessment sheet that contained 12 blocks of photos, each block containing two photo options. Each of the twelve questions had two photo options that the participants could circle: relative to the question, one option corresponded to a positive attitude toward bonobo conservation (pro-conservation option) and the other option corresponded to either a neutral or negative attitude toward bonobo conservation (non-conservation option). We determined which option was pro-conservation or non-conversation based on the messaging and lessons emphasized by the education team during the tour. The questions addressed participants’ attitudes toward the following categories: (1) bonobos as pets, (2) the value of Congolese forest, (3) perceptions of bonobo social behavior, and (4) tendency to objectify or humanize bonobos. This study used a between subject’s design. To control for differences between schools, half of the children in each school were assigned to the *pre-tour* condition and half to the *post-tour* condition. Students in the *pre-tour* condition (*N* = 101) completed the Attitude Assessment at their schools before an in-school information session conducted by the sanctuary’s education staff. In the *post-tour* (*N* = 102) condition, a separate group of students took the Attitude Assessment at Lola ya Bonobo immediately following the guided tour. The order of the photo blocks was determined randomly, and there were two versions of the assessment that counterbalanced the order of the photos within each of the photo blocks. All assessments were anonymous in order to avoid potential experimenter effects.

At the beginning of each week, the education team went to schools to conduct the in-school information session. Before the lesson began, a member of the education team who acted as the experimenter split the classroom into the pre and post-tour groups. The groups were determined by splitting the group in half alphabetically by first name, with the first half being in the pre-tour condition, and the second half in the post-tour condition. Those in the post-tour group were asked to temporarily leave the room while the students took the assessment. Each of the students in the pre-tour group was then given a copy of the survey and a pen. The experimenter stood at the front of the room and first explained the instructions, emphasized that the survey should be taken individually and silently, and emphasized that there were no right or wrong answers. The survey was framed not as an evaluation of conservation attitudes, but as a request for information needed to design advertisements to help Lola ya Bonobo attract more visitors like themselves. Each question corresponded to one of the blocks of photos. While asking the question, the experimenter held enlarged versions of the two photo options to ensure all participants were on the right set of photo options. After asking the question, the experimenter instructed the participants to circle the photo that they thought best answered the posed question. At the end of the 12 questions, the experimenter instructed students to fill out the demographic questions and provided assistance for those who needed it. The assessment was written and conducted in French and approved by Congolese members of the Lola Ya Bonobo Education team for clarity and cultural appropriateness.

The photo options were predetermined as either “pro-conservation” or “non-conservation.” Participants’ responses for each question were scored as “pro-conservation” or “non-conservation” based on which option they marked, circled, dashed, fully underlined or partially underlined. The vast majority of responses unambiguously marked a single answer that could reliably be scored. In the few cases where responses were ambiguous (multiple responses circled), the question was scored as unanswered.

All analyses were conducted in R version 1.0.136 using the glm function. Two analyses were conducted for this study: the first compared the means of the pre-tour and post-tour conditions’ total number of pro-conservation answers. For this overall analysis, we used a generalized linear model (GLM) to analyze whether there was a difference between the mean number of correct responses in the pre-tour and post-tour conditions. Age, gender, and school were included as covariates in this model. For the categorical variables, gender and school, a reference group was pre-determined against which the other groups within the category would be compared. Female was set as the reference group for gender, and Kimbala school was set as the reference group for school.

The second GLM examined the difference between the mean number of correct answers for the pre-tour and post-tour conditions for each individual question. Age, gender, and school were included as predictor variables in the same way they were for the previous analysis. Binomial tests were also conducted to assess whether the mean scores for individual questions were above or below the 50% chance value.

### Attitude Assessment Results

Results from the GLM show that participants chose more pro-conservation options in the post-tour (0.73 ± 0.02) than pre-tour condition (0.68 ± 0.02) (estimate = 0.27, *p* = 0.006) (Figure 1B and Table 2). Participants attending the reference group school (Kimbala) overall answered with significantly more pro conservation responses than participants from the Ngolu school (estimate = −0.432, *p* = 0.001). There was no effect of age or gender.

**TABLE 2:** Descriptive information for participants for all studies.

	Sample	Mean ±		Mean age ±	*M*/*F*
	size	Std. err	Std dev	Std err	ratio
**Study 1: Attitude**					
Pre-Test	101	0.68 ± 0.02	0.15	12.05 ± 0.23	1.12
Post-Test	102	0.73 ± 0.02	0.15	12.73 ± 0.28	0.92
**Study 2: Knowledge**					
Pre-Test	81	0.59 ± 0.0	0.17	23.5 ± 1.24	1
Post-Test	100	0.59 ± 0.02	0.16	20.58 ± 1.03	1.57
**Study 3: Empathy**					
Pre-Test	34	0.48 ± 0.0	0.19	30.57 ± 1.82	0.81
Post-Test	29	0.40 ± 0.03	0.18	34.71 ± 1.67	0.36

Participants responded above chance levels with pro-conservation responses in 7 out of 12 questions in the pre-tour condition and 10 out of 12 in the post-tour condition (see Supplementary Table S3). Subjects scored particularly high (79–99% correct) in at least one condition for five questions (3, 6, 8, 10, and 12) and low (<30% correct) in the pre-tour condition for question 9 (i.e., which group do you think bonobos belong to? Monkeys or humans?).

Examining descriptive statistics (Supplementary Table S3), mean pro-conservation responses increased post-tour in five questions (range: 7–24%), did not change for six and decreased in one (27%). These differences were significant for 8, 9, and 10 in which post-tour correct responses were higher (Question 8: Which photo better shows the value of the forest? Lumber or the standing uncut forest?: estimate = 2.296, *p* = 0.001; Question 9: Which group do you think bonobos belong to? Monkeys or humans?: estimate = 1.211, *p* = 0.017; Question 10: which photo do you think is best for an advertisement about LyB? A photo of Africa or a photo of the DRC?: estimate = 2.496, *p* = 0.029) and for question 2 where correct responses significantly decreased post-tour (Which group do you think bonobos belong to? Wild Animals or Domesticated Animals: estimate = −1.356, *p* = 0.007).

Comparing questions individually across schools again shows that participants in Kimbala School, the reference school, chose more pro-conservation responses than participants from the Mamfufu School (estimate = −1.601, *p* = 0.003) and the Ngolu School (estimate = −1.932, *p* < 0.001).

### Attitude Assessment Discussion

In support of the conservation education hypothesis, our attitude assessment using implicit measures suggests that interactions with bonobos on guided tours at the sanctuary increase pro-conservation attitudes among grade school age children. Overall participants in the post-tour condition selected more of the pro-conservation responses than those in the pre-tour condition, with significant increases in pro-conservation responses in three questions and a decrease in only one.

Four questions asked participants to choose images to use in an advertisement for Lola Ya Bonobo. All showed increases in pro-conservation responses with two being significant increases post-tour. Participants were significantly more likely in the post-tour condition to prefer bonobos being depicted in the wild than in human contact as a pet (Question 10) and were more likely to choose to represent the value of a forest in its natural state rather than as lumber (Question 8).

A subset of questions examined whether participants were likely to humanize or objectify bonobos. In these questions, participants had the option of grouping bonobos with (1) humans or monkeys, (2) humans or vermin, and (3) humans or inanimate objects. The results for these questions are mixed since children were more likely to group bonobos with humans as opposed to with objects or pests, but they were less likely to group bonobos with humans as opposed to with monkeys. Children did shift their preferences to grouping bonobos with humans after the tour, but the mean was still below 50%. This suggests that the tour’s discussion of the genetic, physical, emotional and behavioral similarities shift attitudes in a positive direction, but within limits.

The use of implicit measures designed to reduce experimenter effects was a novel feature of the assessment. Results provide validation for this form of assessment since it largely replicates previous findings using explicit knowledge assessment (André et al., 2008). The use of pictures as choice options also increases the feasibility of assessing attitudes in populations where there are tremendous disparities in literacy levels.

Children at the Kimbala school outperformed children at the Ngolu school and outperformed participants from the Mamfufu school in certain questions. This is perhaps due to proximity—Kimbala is the closest of the four schools to the Sanctuary. Lola Ya Bonobo employs individuals and sources food and supplies from nearby communities. Though none of the participants at Kimbala had previously visited Lola Ya Bonobo before, they perhaps had more awareness of the sanctuary because of friends or families who were employed by the sanctuary.

Overall Lola ya Bonobo’s educational tours have a positive impact on conservation knowledge (André et al., 2008) and attitudes in children (the present study). What is needed next is to understand if similar effects occur in adult visitors, whose choices about whether to engage in the illegal wildlife trade directly impact bonobo conservation.

## Study 2: Knowledge Assessment

The majority of the efforts at Lola Ya Bonobo focus on the education of children and young adults, as they are assumed to be the populations most receptive to conservation messaging. Older individuals also visit the sanctuary but adults may be less open to changes in their beliefs about or attitude toward conservation of endangered species. However, they are responsible for policy changes that influence the future of biodiversity in the DRC. Thus, in study 2, we examined whether adults also learn the core conservation messages that the sanctuary aims to communicate. Study 2 was therefore designed to test how the education program affects knowledge among visitors who are more representative of the general population of Kinshasa in age, economic and educational background.

### Knowledge Assessment Methods

Participants in the Knowledge Assessment were day visitors to Lola Ya Bonobo Sanctuary (*N* = 181, mean age 21.88, age range 8–59; M/F ratio: 93/73). The majority of participants were of Congolese origin (146/181), with the remaining participants of Western European or American origin. Most participants reported being first time visitors to the sanctuary (117/181).

The Knowledge Assessment examined whether visitors to the sanctuary absorbed the main information points emphasized by the sanctuary education program. These points were identified based on observation of the education program in action, and through consultation with the education team. The knowledge assessment was designed to measure what visitors knew about bonobos and facts relating to their conservation. It included twelve true/false and multiple-choice questions, addressing bonobos’ (1) habitat, (2) social organization, (3) similarities to other great apes including humans, and (4) the rehabilitation process for bonobos at the sanctuary. In addition to conservation knowledge questions, we collected demographic information about participants’ age, gender, country of origin, country of residence, and whether or not they had previously visited the sanctuary (see Supplementary Material).

In a between-subjects design, participants completed the questionnaire at the sanctuary either immediately before (pre-tour condition, *N* = 81) or after a guided tour (post-tour condition, *N* = 100). The sanctuary offers four scheduled guided tours each day, 6 days a week. While visitors were waiting for the tour to begin at the Education Center, the guide introduced the optional survey, told visitors that they would receive candy for completing the survey. Each arriving party would randomly be assigned to either the pre-tour condition or the post-tour condition. At the beginning of the tour, the guide handed the surveys, clipboards and pens to participants in the pre-tour group and instructed them to complete the survey individually and silently. He would instruct those in the pre-tour condition to not share anything about the survey with those in the post-tour condition. The pre-tour condition participants had 10–15 min to complete the survey and then the hour-long tour began. Right before the end of the tour, when all participants were back in the Education Center, those in the post-tour condition were given the survey with the same instructions. The assessment was written and conducted in French and approved by Congolese members of the Lola Ya Bonobo Education team for clarity and cultural appropriateness.

Scoring was the same as in Study 1. Like study 1, two analyses were conducted; a GLM that compared the overall number of correct answers between the pre-tour and post-tour conditions which included age group, gender, and number of visits to the sanctuary as covariates. Another GLM was also used to compare the pre-tour and post-tour responses for each individual question, with age group and gender as covariates. Question 3 was used as the reference group for this analysis because there was no difference in mean responses between the two conditions for question 3. All analyses were conducted in R version 1.0.136.

### Knowledge Assessment Results

Overall participants were above chance in their responses in both the pre and post-tour conditions, resulting in no significant difference between these conditions (Figure 1C and Table 2). Participants responded above chance levels with correct responses in 10 out of 12 questions in the pre-tour condition and 9 out of 12 in the post-tour condition (see Supplementary Table S4). Subjects scored particularly high (79–99% correct) in both conditions for five questions (1, 3, 5, 8, and 11) and low (<30% correct) in at least one condition for three questions where chance was 25% (6, 9, and 12).

Mean pro-conservation responses increased post-tour in five questions (range: 6–18%), did not change for six, and decreased in six (1–24%). These differences were significant for question 1 and 4 in which subjects increased correct responses in the post-tour condition. (Question 1: In which country do bonobos live?: estimate = 1.344, *p* = 0.018; Question 4: which of the following is not illegal in the DRC?: estimate = 2.453, *p* = 0.005).

We found no differences between the responses of first-time visitors and returning visitors within or between the two tour groups. Examining age as a variable we did find that those in the post-tour condition in age group 2 (ages 16–18) made more correct responses than other participants across conditions (estimate = 1.414, *p* = 0.011).

### Knowledge Assessment Discussion

Unlike the prediction of the Conservation Education Hypothesis, composite scores did not increase from the pre-tour assessment to the post-tour assessment. However, in line with this prediction, we did find significant increases in correct answers on two questions. Moreover, the lack of overall difference between conditions likely comes from the high number of correct answers on the assessment in both the pre-test and post-test conditions.

Adult visitors came to the sanctuary with a high baseline level of knowledge about bonobos as reflected in their pre-tour assessment scores. It is important to highlight that given that this population is choosing to visit the sanctuary and paying the entrance fee, they are likely of a higher socio-economic status and education level than the average Congolese citizen. There was no increase or decrease in mean scores in the post-tour condition. The high baseline scores perhaps stem from widescale efforts of programs like Lola Ya Bonobo to disseminate information about bonobos in schools and communities over the past 20 years.

Questions on this knowledge assessment fell into one of two categories: natural history of bonobos and conservation of bonobos. Participants scored well above chance in both conditions for all except three questions (6, 9, and 12). Question 6 (What is the social organization of bonobos?) and Question 9 (Which of the following is least related to a bonobo?) were natural history questions. Both of these questions may have been too detailed for visitors to have known before visiting the sanctuary. Incongruity between the tour guides conveying the answers to these questions and visitors’ observations of the bonobos may have led to confusion of the right answer. Question 12 (Why should we save bonobos) was a conservation related question and may have been perceived as subjective to visitors (the correct answer was *d*: all of the above).

Question 1 (Where are bonobos found?), a natural history question, and Question 4 (Which of the following are not a threat to bonobos), a conservation question had significantly more correct answers in the post-tour compared to the pre-tour condition. Despite having high baseline scores, the results from these two questions support the Conservation Education Hypothesis.

Given the high level of knowledge about bonobos among this population, we next explored whether high levels of pro conservation attitudes existed among a similar subset of adults.

## Study 3: Empathy Assessment

Finding little evidence that the guided tour has little effect on the visitor’s knowledge about bonobos and their conservation, we next examined if the tour impacted visitors’ empathy toward the species. We again wanted to assess the effect of the tour on empathy in the general population of Kinshasa who are representative of the current policy decision makers in the DRC. It is commonly thought that to increase support and interest in species’ conservation, we must increase empathy for the species (Schultz, 2000; Sheeder and Lynne, 2011); however, whether or not conservation programs actually engender empathy in their visitors has not been thoroughly examined (Berenguer, 2007; Sevillano et al., 2007; Tam, 2013). This final study examined whether the guided tour at Lola Ya Bonobo increased visitors’ empathy toward bonobos. We used a novel paradigm using implicit measures to assess empathy in the general population that visited the sanctuary. Our implicit measure for this study was the use of *mentalistic language*, as opposed to *descriptive language*, as a measure of empathy. Mentalistic language describes the internal thought processes of an individual, whereas descriptive language describes the apparent actions of the individual. Bonobos have been shown to have complex social cognitive capacities previously only ascribed to humans (Krupenye et al., 2016, 2017, 2018; Krupenye and Hare, 2018; Krupenye and Call, 2019). Evidence from developmental and social psychology suggest that using mentalistic language to attribute such an inner life to others (e.g., she *feels* happy as opposed to she *looks* happy) is an indication of an individual’s ability to understands the internal thoughts of others, and consequently is a trait commonly thought to underlie the ability to empathize with others (Ruffman et al., 2002; Symons, 2004). The use of mentalistic language as a measure of empathy has been examined among groups of humans, both adult and children (Ruffman et al., 2002). This study is the first to examine attribution of mentalistic language between humans and an endangered species.

### Empathy Assessment Methods

Like the Knowledge Assessment, participants for the Empathy Assessment were adult day visitors to Lola Ya Bonobo (*N* = 63). Among those who recorded their age and gender, the mean age was 31.95 years and the male/female ratio was 27/17. Only 39 participants responded to the question about whether or not they had previously visited the sanctuary. Of those 39 participants, 28 participants indicated it was their first visit. The procedure for this study was identical to the procedure for the Knowledge Assessment.

This assessment examined whether the experience of seeing and interacting with the bonobos at the sanctuary increased visitors’ empathy for the bonobos. We examined the use of *mentalistic language* as a measure of empathy. All survey materials were in French. The survey consisted of six photos of bonobos doing various actions like eating, playing, pointing, or sitting. Underneath each photo were two options that described what was happening in the photos—one option used mentalistic language and the other option used descriptive language. The survey instructed participants to choose which of the two options best described what was happening in the photo. In addition to the six questions, we also collected information about participants’ age, gender, country of origin, country of residence, and whether or not they have previously visited the sanctuary (see Supplementary Material). There were two versions of the survey that counterbalanced whether the mentalistic or descriptive option was displayed first. The two different versions were randomly distributed among participants.

For this between-subject design, participants in the *Pre-Tour* condition (*N* = 34) completed the Empathy Assessment at Lola Ya Bonobo before the start of the guided tour and those in the participants in the *Post-Tour* condition (*N* = 29) took the assessment immediately following the guided tour. The assessment was written and conducted in French and approved by Congolese members of the Lola Ya Bonobo Education team for clarity and cultural appropriateness.

Scoring for this study used the same criteria as those in previous studies. One challenge we encountered in this study was that not enough participants filled out the demographic information to use any of the demographics as covariates.

For the overall analysis, the same GLM was used as in the attitude assessment. Binomial tests were also conducted to assess whether the mean scores for individual questions were above or below the 50% chance value. We used the available but incomplete demographic data to test the effect of age and gender.

### Empathy Assessment Results

There was not an overall significant difference between the pre- and post-tour groups (Figure 1D and Table 2). Participants responded above chance levels with empathy responses in two out of six questions in the pre-tour condition and one out of six in the post-tour condition (see Supplementary Table S5). Subjects did not score particularly high in any of the six questions but scored low (<30% pro-empathy) in at least one condition for three questions out of six (3, 5, and 6). There was no apparent effect of age or gender. Like the Knowledge Assessment, the sample population for this study is not representative of all of Kinshasa. Because they are choosing to visit the sanctuary and pay the entrance fee, this study population is likely wealthier and more educated than the average Congolese citizen.

Mean pro-empathy responses increased post-tour in one question (8%) and decreased in five (1–29%). None of these differences were significant for individual questions. Although not significant, the post-tour group showed a 26 and 29% drop, respectively, in empathic responses after the tour in question two and six.

### Empathy Assessment Discussion

Results from the Empathy Assessment do not provide further support the Conservation Education Hypothesis. Question 4 was the only question where participants in the post-tour condition chose the mentalistic language above chance. However, this study may not have been sufficiently sensitive to capture positive changes in empathy. Further research should investigate other potential implicit attitude assessments among adults, especially those that explore changes in empathy toward animals, and should attempt to calibrate assessments to prevent such ceiling effects in pretest results. Using mentalistic language as an implicit measure of attitude toward species is still novel. Further exploration of mentalistic language and other implicit measures is needed to best assess changes in adults’ attitudes toward bonobo conservation in this population.

## General Discussion

Building upon the previous knowledge assessments conducted at Lola Ya Bonobo in 2008, our results in children support the Conservation Education Hypothesis. There is less support for the hypothesis among adult visitors.

An assessment conducted in 2008 supported the prediction that the education tour at Lola Ya Bonobo sanctuary positively impacted children’s conservation knowledge of bonobos. The Attitude Assessment described in study 1 further demonstrates that children are likely to have stronger pro-conservation attitudes toward bonobos after the sanctuary tour compared to before the tour. Specifically, results suggest that the tour reinforces children’s belief that bonobos are not appropriate pets and that the forest habitat of bonobos has inherent value. This advance is important because it suggests, while controlling for experimenter demand effects, that children may internalize and update their own personal views as a result of the pro-conservation teaching offered by the sanctuary. Moreover, this paradigm offers a new tool for assessing implicit changes in attitudes in a wide range of populations with varying degrees of literacy.

The Knowledge Assessment (Study 2) and Empathy Assessment (Study 3) did not provide strong support for the CEH. However, they contributed important insights by examining the tour’s influence on pro-conservation knowledge and attitudes in adults, a critical population that had not been previously studied at this sanctuary. Study 2 found that adults came into the tour with a high level of knowledge about bonobos and their conservation. There was no significant overall change in level of knowledge after the tour, although adults showed significant improvements on question 1 (In which countries do bonobos live?) and question 4 (which of the following is not illegal in the DRC?). Although these results may suggest that the tour itself did not dramatically impact adult visitors’ knowledge about bonobos and their conservation, it may be that the education outreach the sanctuary has conducted over the past 20 years has raised baseline levels of knowledge among the general population of Kinshasa in the Democratic Republic of the Congo. The null results for the Empathy Assessment (Study 3) suggest that further research is needed on how to best assess conservation attitude change among this population of adults. A future study that includes a larger sample size of Congolese participants would also further illuminate what best influences attitude changes.

In addition to the support for the Conservation Education Hypothesis, the novel methods used in the Attitude Assessment highlight the importance of developing implicit attitude assessments that can be implemented with a wide variety of populations. Using implicit studies that do not heavily rely on language will allow for more objective quantitative comparison of different populations that play a role in the future of great apes.

Even though the analyses of the composite results for the Knowledge Assessment and Empathy Assessment do not strongly support the conservation education hypothesis, certain questions from across all three studies provide important feedback on how the sanctuary can refine its tour to best encourage pro-conservation attitudes. Well-intended messages conveyed by the program could have unintended effects on the audience. For instance, the tour heavily emphasizes the evolutionary relationships between bonobos and humans. Results from Question 9 (Which of the following are bonobos least related to?) in the Knowledge Assessment reveals that participants perform at or below chance on a question related to this topic both before and after the tour. This might be due to religious and cultural beliefs conflicting with the sanctuary’s emphasis on scientific-based messages. Given this result, the education team can experiment on whether or not decreasing the focus on bonobos’ genetic and behavioral similarity to humans will lead to increased pro-conservation attitudes among adult visitors. This example highlights how results from individual questions should be scrutinized under both cultural and educational frameworks to improve the outcomes of a conservation-minded program. Conservation education program evaluations should be adapted to the cultural norms and cultural practices of the program’s target populations.

Our research here is limited in several ways. We used a between-subject design as opposed to a within-subject design. These studies were designed to be between subject primarily to reduce demand on participants. Additionally, instructing participants that they will take an assessment both before and after the tour ay encourage participants to attend to information in a way that is not representative of the average visitor experience.

In the Attitude Assessment, implicit attitude measures conducted with children are not concurrently compared to the results of a more conventional explicit assessment of attitude. To account for this, we relied on qualitative comparisons to the limited attitude assessment conducted among children in André et al. (2008).

The Knowledge Assessment was limited to adults who voluntarily visited the sanctuary. Voluntarily coming to the sanctuary suggests pre-existing interest in learning about bonobos and their conservation. Additionally, the entrance fee ($5 USD) is higher than the average wage/day in Kinshasa ($2 USD/day), which suggests that the population visiting the sanctuary is considerably more middle-class than the average individual in the DRC. This middle-class population of Kinshasa though not fully representative of the DRC, mirrors sanctuary and zoo-going visitors in other countries that have been more thoroughly studied. The knowledge assessment was indeed the only study in this series that did not involve implicit measures. It was targeted toward the more educated, middle class population visiting the sanctuary among whom literary levels and experience with surveys was higher than the average population in Kinshasa. Although participants generally performed well on the Knowledge Assessment, future work could simplify questions that proved difficult and adapt the questionnaire for a more diverse sample.

The Empathy Assessment is limited because it may not have assessed attitudes pertinent to this Congolese population. The emphasis on empathy toward animals may be culturally dependent (Paul, 2000). In more industrialized and Western countries, animals are more likely to co-habitate with humans and are seen as part of a family (Negra and Manning, 1997; Daly and Suggs, 2010). This proximity breeds stronger feelings of empathy toward animal more generally. This attitude may contrast with conventional attitudes toward animals in Central Africa, where animals are viewed in more utilitarian ways and as belonging to a domain distinctly separate from humans. For this Congolese population, viewing bonobos in a semi-wild habitat may highlight exactly how different they are from humans. The experience of the tour may in some ways counteract the sanctuary’s desire to increase empathy for bonobos among adult visitors. Further cross-cultural research is needed to understand the different role empathy plays in cultivating pro-conservation attitudes toward species like bonobos.

As it relates to all three studies, assessments were only conducted directly after the tour. Future studies will need to develop innovative and implicit methods of assessing how educational interventions, such as guided sanctuary tours, affects both children’s and adults’ changes in knowledge, attitude, and behavior in the days, weeks, and months that follow.

Despite limitations, the Conservation Education Hypothesis provides a useful framework for exploring the effectiveness of environmental education programs. Activities like poaching as well as climate change resulting from human behavior have caused mass species extinction. Some species, like the three species of African Great Apes, are currently being pushed toward extinction because of human behavior. It is crucial that conservation organizations allocate their finite resources toward programs that demonstrate effective change for wildlife conservation. These organizations can improve their outcomes by employing recommendations from evidence-based research. Testing the Conservation Education Hypothesis is one way organizations can explore whether their education programs are effectively changing conservation attitudes and behaviors.

The results from these three studies underscore the important role wildlife sanctuaries play in changing the knowledge and attitudes of their visitors. In particular, this study highlights the importance of focusing on communities that have major influence on the future of endangered species. The population of the DRC drives the greatest demand for bushmeat in the Congo Basin (Wilkie and Carpenter, 1999), but Congolese populations have not been extensively studied regarding the drivers that influence attitudes toward wildlife. Future studies with this population should consider the local cultural histories and beliefs in order to design new communication strategies that can most effectively lead to pro-conservation attitudes. A reliance on empathy, while proposed to be an effective strategy for generating attitude and behavior change in more Westernized cultures, may not be an effective communication strategy or measure of change in this community. Alternatively, future studies with this population could explore additional ways to test changes in empathy toward bonobos that were not explored in the present set of studies.

Children play an important role in influencing their peers and family members to pursue more pro-conservation behaviors. Since Lola Ya Bonobo’s inception, a significant number of phone calls about bonobos as pets in need of rescue have come from children who have previously visited the sanctuary (André et al., 2008). Children play an important role in wildlife conservation, but adults have the agency to stall imminent threats of species extinction and climate change. Therefore, in addition to further work on children, future studies should focus on behavior change in adult populations. Though the results can only be qualitatively compared due to differences in method, these set of studies found significant changes in attitudes in children but not in adults. Future studies should focus on social, economic, and political reasons why conservation education experiences are less likely to shift pro-conservation attitudes in adults, while incorporating more direct comparisons between children and adults. Determining ways to encourage pro-conservation attitudes and behaviors from childhood through adulthood is ultimately how conservation education programs can help reverse the threats of wildlife extinction.

## Data Availability Statement

All datasets generated for this study are included in the article/Supplementary Material.

## Ethics Statement

Ethics approval for all studies was granted by the Duke University Campus IRB protocol #D0939. Approval was granted as a secondary analysis of data originally collected by the Lola Education team for program improvement purposes.

## Author Contributions

AB, CK, and BH designed the studies. AB analyzed the data and wrote the manuscript. AB and CK conducted the studies. PM and FM supported study implementation at Lola ya Bonobo.

## Conflict of Interest

FM and PM were employed by the Lola Ya Bonobo. The remaining authors declare that the research was conducted in the absence of any commercial or financial relationships that could be construed as a potential conflict of interest.
