# Supplementary material for: Implicit Measures Help Demonstrate the Value of Conservation Education in the Democratic Republic of the Congo
Source: Front Psychol. 2020 Mar 13;11:386. doi: 10.3389/fpsyg.2020.00386 (PMC7083138; doi:10.3389/fpsyg.2020.00386)
Supplement: Supplementary file 1 [file Table_1.docx]

Supplementary Material

Supplementary Table 1: Results of study 1 on Attitude Assessment in children. Means for the pre-test and post-test represent the number of individuals who correctly answered the question divided by the total number of individuals who answered the question. Asterisks (*) indicate that the proportion of individuals answering correctly (pro-conservation) is above chance level (p<0.05).

| **Question** | **Pre-Test Mean** | **Post Test Mean** | **GLM Result** |
| --- | --- | --- | --- |
| 1. Which of these photos would you prefer to see on an advertisement for Lola Ya Bonobo? (Pet or Wild?) | .445 | .604 | NS |
| 2. Which group do you think bonobos belong to? (Wild Animals or Domesticated Animals) | *.723 | .449 | Estimate=-1.356, p=.007 |
| 3. Which group do you think bonobos belong to? (Apes or Monkeys?) | *.792 | *.832 | NS |
| 4.Which pictures reminds you more of bonobos? (Yogis or soldiers?) | .667 | .68 | NS |
| 5. Which of these photos would you prefer to see on an advertisement for Lola Ya Bonobo? (Pet or Wild?) | .64 | *.772 | NS |
| 6.Where would you prefer to find bonobos? (In a market or in a forest?) | *.98 | *.98 | NS |
| 7. Which group do bonobos belong to? (Humans or pests?) | *.717 | *.727 | NS |
| 8. Which photo better shows the value of the forest? (Lumber or the standing uncut forest?) | *.71 | *.95 | Estimate=2.296, p=.001 |
| 9. Which group do you think bonobos belong to? (Monkeys or humans?) | .198 | .4 | Estimate=1.211, p=.017 |
| 10. Which photo do you think is best for an advertisement about LyB? (A photo of Africa or a photo of the DRC?) | *.901 | *.99 | Estimate=2.496, p=.029 |
| 11. Which group do you think bonobos belong to? (Humans or objects?) | .465 | .534 | NS |
| 12. Which of these photos would you prefer to see on an advertisement for Lola Ya Bonobo? (Pet or Wild?) | *.921 | *.931 | NS |

Supplementary Table 2: Results of study 2 on Knowledge Assessment in adults. Means for the pre-test and post-test represent the number of individuals who correctly answered the question divided by the total number of individuals who answered the question. Asterisks (*) indicate that the proportion of individuals answering correctly is above chance level (p<0.05).

| **Question** | **Chance Level** | **Pre-Test Mean** | **Post Test Mean** | **GLM Result** |
| --- | --- | --- | --- | --- |
| 1. In which countries do bonobos live? | .25 | *.838 | *.99 | estimate=1.344, p=.018 |
| 2. Which of the following is NOT a threat to bonobos? | .25 | *.597 | .*659 | NS |
| 3. T/F: Bonobos don’t suffer when they are killed. | .5 | *.851 | *.849 | NS |
| 4. Which of the following is NOT illegal in the DRC? | .25 | *.474 | *.607 | (estimate=2.453, p=.005). |
| 5. True or False: Unlike humans, bonobos don’t need the care of their mothers to survive. | .5 | *.885 | *.826 | NS |
| 6. Which of the following describes the social organization of bonobos? | .25 | *.48 | .239 | NS |
| 7. Which of the following is NOT true about eating bonobos? | .25 | *.532 | *.5 | NS |
| 8. T/F: Bonobos feel many of the same emotions as humans. | .5 | *.81 | *.989 | NS |
| 9. Of the species listed below, which is the LEAST like bonobos? | .25 | .197 | .277 | NS |
| 10. What should you do if you see a bonobo being sold in the market? | .25 | *.734 | *.73 | NS |
| 11. T/F: Baby bonobos are traumatized when poachers take them from their families. | .5 | *.938 | *.928 | NS |
| 12. Why should we save bonobos? | .25 | .342 | .269 | NS |

Supplementary Table 3: Results of study 3 on Empathy Assessment in adults. Means for the pre-test and post-test represent the number of individuals who correctly answered the question divided by the total number of individuals who answered the question. Asterisks (*) indicate that the proportion of individuals answering correctly (with empathy) is above chance level (p<0.05).

| **Question** | **Pre-Test Mean** | **Post Test Mean** | **GLM Result** |
| --- | --- | --- | --- |
| 1. The bonobos are playing./**The bonobos are having fun.** | .424 | .345 | NS |
| 2. The bonobo extends his finger./**The bonobo wants to touch.** | *.71 | .448 | NS |
| 3. **The mother bonobo loves her baby.**/The mother bonobo kisses her baby. | .265 | .345 | NS |
| 4. The bonobo is facing right./ **The bonobo sees something to his right.** | *.706 | *.69 | NS |
| 5. The bonobo eats the food./**The bonobo is hungry and wants the food.** | .206 | .172 | NS |
| 6. **The orphan bonobo in the corner of the box is scared./**The orphan bonobo is in the corner of the box. | .559 | .269 | NS |

**Study 1: Attitude Assessment Script and Materials**

An experimenter asked participants the following questions either before or after they toured the sanctuary and participated in the education program. Experimenters read the questions, said aloud two potential answers (*in parentheses*) and then allowed subjects to indicate their answer by circling the corresponding photo on the answer sheet. The pro-conservation answer is marked with a red “1” on the answer sheet.

| 1. Which of these photos would you prefer to see on an advertisement for  Lola Ya Bonobo?  (*Pet or Wild?)* |
| --- |
| 2. Which group do you think bonobos belong to? *(Wild Animals or*  *Domesticated Animals)* |
| 3. Which group do you think bonobos belong to? *(Apes or Monkeys?)* |
| 4.Which pictures reminds you more of bonobos? *( People praying or soldiers?)* |
| 5. Which of these photos would you prefer to see on an advertisement for  Lola Ya Bonobo?  (*Pet or Wild?)* |
| 6. Where would you prefer to find bonobos? *(In a market or in a forest?)* |
| 7. Which group do bonobos belong to? *(Humans or pests?)* |
| 8. Which photo better shows the value of the forest?  *(Lumber or the standing uncut forest? )* |
| 9. Which group do you think bonobos belong to? (*Monkeys or humans?)* |
| 10.Which photo do you think is best for an advertisement about LyB?  *(A photo of Africa or a photo*  *of the DRC?)* |
| 11.Which group do you think bonobos belong to? *(Humans or objects?)* |
| 12. Which of these photos would you prefer to see on an  advertisement for Lola Ya Bonobo?  (*Pet or Wild?)* |

**Study 2: Knowledge Assessment Materials (translated from original French)**

**Study 3: Empathy Assessment Materials**

Translation of materials are below image. The pro-empathy response is indicated with a red “1”.

| 1. The bonobos are playing./**The bonobos are having fun.** |
| --- |
| 2. The bonobo extends his finger./**The bonobo wants to touch.** |
| 3. **The mother bonobo loves her baby.**/The mother bonobo kisses her baby. |
| 4. The bonobo is facing right./ **The bonobo sees something to his right.** |
| 5. The bonobo eats the food./**The bonobo is hungry and wants the food.** |
| 6. **The orphan bonobo in the corner of the box is scared./**The orphan bonobo  is in the corner of the box. |
|  |
